# Decitabine Inhibits Gamma Delta T Cell Cytotoxicity by Promoting KIR2DL2/3 Expression

**DOI:** 10.3389/fimmu.2018.00617

**Published:** 2018-03-26

**Authors:** Chao Niu, Min Li, Shan Zhu, Yongchong Chen, Lei Zhou, Dongsheng Xu, Wei Li, Jiuwei Cui, Yongjun Liu, Jingtao Chen

**Affiliations:** ^1^Department of Translational Medicine, The First Hospital of Jilin University, Changchun, China; ^2^Department of Cancer Center, The First Hospital of Jilin University, Changchun, China; ^3^Sanofi Research and Development, Cambridge, MA, United States

**Keywords:** DNA methyltransferase inhibitor, adoptive cell immunotherapy, DNA methylation, killer Ig-like receptors, myelodysplastic syndrome

## Abstract

Gamma delta (γδ) T cells, which possess potent cytotoxicity against a wide range of cancer cells, have become a potential avenue for adoptive immunotherapy. Decitabine (DAC) has been reported to enhance the immunogenicity of tumor cells, thereby reinstating endogenous immune recognition and tumor lysis. However, DAC has also been demonstrated to have direct effects on immune cells. In this study, we report that DAC inhibits γδ T cell proliferation. In addition, DAC increases the number of KIR2DL2/3-positive γδ T cells, which are less cytotoxic than the KIR2DL2/3-negative γδ T cells. We found that DAC upregulated KIR2DL2/3 expression in KIR2DL2/3-negative γδ T cells by inhibiting *KIR2DL2/3* promoter methylation, which enhances the binding of *KIR2DL2/3* promoter to Sp-1 and activates *KIR2DL2/3* gene expression. Our data demonstrated that DAC can inhibit the function of human γδ T cells at both cellular and molecular levels, which confirms and extrapolates the results of previous studies showing that DAC can negatively regulate the function of NK cells and αβ T cells of the immune system.

## Introduction

Over the last decade, adoptive cell immunotherapy using gamma delta (γδ) T cells, which can secrete abundant cytokines and exert potent cytotoxicity against different cancer cells, including solid tumor cells (1–3) and hematological tumor cells, has shown high therapeutic potential in cancer treatment (4, 5). γδ T cells are a unique subset of lymphocytes that express T cell receptors composed of γ and δ chains. Although γδ T cells constitute a very small percentage of human lymphocytes, they are widely distributed in the peripheral blood and mucosal tissues (6, 7). γδ T cells rapidly recognize exogenous pathogens and endogenous stress-induced ligands in a major histocompatibility complex (MHC)-unrestricted manner and initiate adaptive immunity, thereby acting as a first line of immune defense (8–10). Many clinical trials have been conducted in an effort to harness the abilities of γδ T cells and test the efficacy of γδ T cells in adoptive immunotherapy (11–13). γδ T cells have become a potential avenue for adoptive cell immunotherapy (14, 15).

The epigenetic drug decitabine (DAC) is approved for the treatment of myelodysplastic syndrome (MDS) and has been introduced for the treatment of acute myeloid leukemia (AML). In the past two decades, the clinical benefits of DAC in solid tumors have been reported (16, 17). DAC displays a dual mechanism of action depending on the dose, i.e., reactivation of silenced genes as well as differentiation at low doses, and cytotoxicity at high doses (18–20). DAC is a DNA methyltransferase inhibitor that induces the re-expression of silenced genes such as tumor suppressor and cell cycle-regulating genes, thereby resulting in increased apoptosis or decreased proliferation of tumor cells (21–23). Recently, preclinical studies have indicated that DAC enhanced the expression of cancer-testis antigens, the death receptor FAS, MHC class I molecules, and co-stimulatory molecules, and boosted the cytolytic activity and proliferation of T cells (24–28). DAC plays an immune stimulatory role in cancer therapy by sensitizing tumor cells to immune responses. Therefore, recent clinical studies are utilizing DAC prior to or in combination with immune therapies to improve clinical outcomes (29). However, DAC can inhibit the antitumor functions of immune cells, including lysis of tumors by NK cells, naïve αβ T cell proliferation, and increasing the number of regulatory T cells (20, 30, 31).

In this study, we show that DAC inhibits γδ T cell proliferation. At the molecular levels, DAC increased the number of KIR2DL2/3-positive γδ T cells, which are less cytotoxic than KIR2DL2/3-negative γδ T cells. We found that DAC upregulated KIR2DL2/3 expression in KIR2DL2/3-negative γδ T cells by inhibiting *KIR2DL2/3* promoter methylation, which enhances the binding of *KIR2DL2/3* promoter to Sp-1 and activates *KIR2DL2/3* gene expression. Therefore, we suggest that DAC may represent a double edged sword in the immune system that stimulates antitumor immunity by promoting tumor antigen presentation and costimulation, and inhibits antitumor immunity by blocking the function of NK cells, αβ T cells, and γδ T cells.

## Materials and Methods

### Patient Enrollment

Seven newly diagnosed MDS and AML patients who did not receive radiation therapy and chemotherapy before blood collection were enrolled in this study, all of whom provided written informed consent for the use of biospecimens for research purposes in accordance with the Declaration of Helsinki. The study was approved by the Ethics Committee of the First Hospital of Jilin University and carried out in accordance with the approved guideline “Use of experimental animals and human subjects.” The patient information is shown in Table 1.

**Table 1:** Patient clinical characteristics.

Patient no.	Gender	Age	Type of tumor	Stages of disease
1	Male	50	Acute myeloid leukemia (AML)	M3
2	Male	30	AML	M3
3	Male	80	Myelodysplastic syndrome (MDS)	RCMD
4	Male	75	MDS	RAEB-2
5	Male	69	MDS	RAEB-2
6	Male	56	MDS	RAEB-2
7	Male	43	MDS	RAEB-2

### Protections for Lab Personnel

To protect our laboratory personnel from infections, we did not collect blood samples from patients infected with HIV, HBV, or HCV. Blood sample processing was performed in a biosafety cabinet. Operators had to wear sterile gloves and masks while handling blood samples.

### Pharmacological Agents

DAC was kindly provided by China Tai Tianqing Pharmaceutical Group Co., Ltd. (CTTQ) and dissolved in saline to a concentration of 10 mM. Zoledronate, which serves as an antigen to stimulate and expand γδ T cells, was purchased from Jilin Province Xidian Pharmaceutical Sci-Tech Development Co., Ltd.

### Cell Culture

The human MDS cell line, SKM-1, Burkitt’s lymphoma cell line, Raji (NK-resistant), and AML cell line, K562 (NK-sensitive) were kindly provided by Dr. Jifan Hu at Stanford University Medical School, Palo Alto Veterans Institute for Research, Palo Alto, CA, USA. All cell lines were cultured in RPMI-1640 medium (Gibco, Grand Island, NY, USA) supplemented with 10% heat-inactivated fetal bovine serum (FBS; Gibco), 100 U/mL penicillin, and 100 mg/mL streptomycin (Invitrogen, Carlsbad, CA, USA) at 37°C in a humidified 5% CO_2_ incubator.

### Expansion of γδ T Cells

γδ T cells were expanded as previously reported (32, 33). Briefly, heparinized peripheral blood samples were obtained from seven MDS and AML patients. Blood samples were centrifuged at 1,800 × *g* for 10 min, and the plasma was transferred to new tubes. Peripheral blood mononuclear cells (PBMCs) were isolated by density gradient centrifugation using Ficoll (Nycomed Pharma AS, Oslo, Norway) at 800 × *g* for 30 min. To expand γδ T cells, PBMCs were cultured in AIM-V medium CTS™ (Gibco) with 1 µM zoledronate, 5% auto-plasma, and 500 U/mL human IL-2 (Miltenyi Biotec GmbH, Bergisch Gladbach, Germany) for 9 days. Fresh complete medium with IL-2 supplement (500 U/mL) was added every 2 or 3 days. The cultured cells were expanded γδ T cells and treated with DAC without sorting. KIR2DL2/3^+^ and KIR2DL2/3^−^ γδ T cells were sorted from these cultured cells using a flexible BD Influx™ cell sorter (BD Biosciences, San Jose, CA, USA).

### Proliferation Assay

Expanded γδ T cells (1 × 10^6^ cells/mL) were incubated and stained with 1 µM carboxyfluorescein succinimidyl ester (CFSE) (Molecular Probes, Eugene, OR, USA) according to the manufacturer’s recommended protocol. The labeled cells were then washed, suspended (1 × 10^6^ cells/mL), and incubated with increasing doses (0, 0.25, 0.5, 1, 2, 3, 4, and 5 µM) of DAC at 37°C in 5% CO_2_. After incubation for 5 days, the cells were collected and stained with Vγ9-PE (BD Biosciences). After staining, the cells were analyzed using a BD FACS Calibur (BD Biosciences) with Cell Quest Pro software, and the final analysis was performed using FlowJo software (Tree Star, Ashland, OR, USA).

### Cell Viability Assay

Expanded γδ T cells (1 × 10^6^ cells/mL) were incubated with various concentrations (0, 0.25, 0.5, 1, 2, 3, 4, and 5 µM) of DAC for 48 h. The proportions of living, dead, and apoptotic cells were determined using an Annexin V and 7-AAD staining kit (eBioscience, San Diego, CA, USA) according to the manufacturer’s protocol. After staining, the cells were analyzed using the BD FACS Calibur.

### Cell Cycle Assay

After treatment with various concentrations of DAC, expanded γδ T cells were fixed with cold 70% ethanol overnight at −20°C, followed by washing once with cold phosphate-buffered saline (PBS). The fixed cells were treated with RNase and stained with propidium iodide (Sigma-Aldrich, St. Louis, MO, USA). The stained cells were analyzed by flow cytometry using ModFit LT software (Verity Software House, Topsham, ME, USA).

### Surface Marker Detection

Expanded γδ T cells treated with 0.5 µM DAC for 48 h were stained with DNAM-1-PE (559789), NKG2D-APC (558071), Vγ9-FITC (555732), KIR2DL2/3 (CD158b)-PE (559785), CD3-PerCP (347344), KIR2DL1 (CD158a)-PE (556063) (BD Biosciences), CD279-APC (329908) (BioLegend, San Diego, CA, USA), KIR2DS4 (CD158i)-APC (FAB1847A), and KIR3DL1 (CD158e1)-APC (FAB1225A) (R&D Systems, Minneapolis, MN, USA). Appropriate isotype-matched antibodies (Abs) were used as controls. Data were analyzed by flow cytometry.

### Cytotoxicity Assay

A calcein-AM release assay was used as previously described to assess cytotoxicity (34). Tumor cells were labeled with 3.5 µM of calcein-AM (Dojindo Laboratories, Kumamoto, Japan)/10^6^ cells/mL and incubated in a humidified incubator at 37°C with 5% CO_2_ for 30 min. After washing twice with PBS, the target cells were adjusted to a concentration of 5 × 10^4^ cells/mL with 5% FBS RPMI-1640 medium and seeded in 96-well plates. The ratio of effector to target cells was set at 10:1. DAC (0.5 μM)-treated γδ T effector cells (100 µL) and untreated cells were added to the target cells in a 96-well plate at the indicated ratio and incubated at 37°C for 4 h. For the KIR2DL2/3 Ab-blocking experiment, the effector cells were first treated with or without Abs to KIR2DL2/3 (312602) (BioLegend, San Diego, CA, USA) for 30 min and then incubated with target cells. After incubation, the supernatant was harvested and transferred to a new plate. Absorbance at 485 nm of excitation light wavelength and 528 nm of emission wavelength was assessed using a BioTek Synergy HT Microplate Reader (BioTek Instruments, Winooski, VT, USA). The specific lysis was calculated according to the following formula: [(experimental release − spontaneous release)/(maximum release − spontaneous release)] × 100%. Spontaneous release was obtained by incubating the target cells in medium alone, and maximum release was obtained after treatment with 1% Triton X-100. All experiments were performed in triplicate wells and at least three independent experiments were completed.

### DNA Methylation by Bisulfite Sequencing

Genomic DNA from γδ T cells treated with and without DAC was isolated using a DNeasy^®^ Blood and Tissue kit (Qiagen GmbH, Hilden, Germany) and modified with bisulfate using a EZ DNA Methylation-Gold™ kit (Zymo Research, Irvine, CA, USA), according to the manufacturer’s instructions. DNA was amplified using specific primers (*KIR2DL2/3* forward: TTGGGTTTTATGTAAGGTAGAAAGAGT; *KIR2DL2/3* reverse: CCAAACCTATATCTCCAACTCTAAAC; *NKG2D* forward: GTGGAGAGGTTAGGTTATTTTTTAA; *NKG2D* reverse: TTACCTCACTCTAAACTTTCACAAAAC). The PCR products were cloned using a pJET PCR Cloning kit (K1231, Thermo Scientific, Waltham, MA, USA), and 12 independent clones from each sample were sequenced to determine DNA methylation status (34).

### Chromatin Immunoprecipitation (ChIP) qPCR Assays

Chromatin immunoprecipitation assays were performed using a Pierce Agarose Chip Kit (Thermo Scientific) according to the manufacturer’s instructions. Specific anti-trimethyl-H3K4 (Cell Signaling Technology, 9727) and anti-Sp-1 Abs (Invitrogen, PA5-29165) were used to determine the promoter methylation profile of KIR2DL2/3. Normal rabbit IgG was used as a negative control. DNA was extracted and analyzed by quantitative real-time PCR (qPCR) with specific primers (Site I forward: ACGTGCTATTCCACCTTTCCT; Site I reverse: CCGGAAGCCTTAGGCAAGAA; Site II forward: GAGACAGTCTCACTCTCTCAC; Site II reverse: AGTTCAAGACCAGCTGGTCCA; Site III forward: GCAGGGCGCCAAATAACATC; Site III reverse: GATGCCCTTCCAGGACTCAC). Enrichment was calculated using the following formula: Relative Enrichment Fold = 2^−[(Ct (DAC group) − (Ct (Input of DAC group) − Log2 (Input Dilution Factor of DAC group))) − (Ct (Control group) − (Ct (Input of Control group) − Log2 (Input Dilution Factor of Control group)))]^.

### Quantitative Real-Time Reverse Transcription PCR (qRT-PCR)

Total RNA was extracted from γδ T cells treated with DAC using an EasyPure RNA kit (Beijing TransGen Biotech Co., Ltd., Beijing, China) according to the manufacturer’s instructions. After removing genomic DNA contamination with DNase I (Sigma-Aldrich), M-MLV Reverse Transcriptase (Invitrogen) was used to synthesize cDNA. KIR2DL2 and KIR2DL3 mRNA expression was quantified by qRT-PCR using a CFX384TM Real Time system (Bio-Rad, Jurong East, Singapore). The PCR reactions were set up in a final volume of 10 µL with 5 µL SYBR Green qPCR Mix (Roche, Indianapolis, IN, USA) and 10 pmol of each sense and antisense primer. The primer sequences were *KIR2DL2* forward: ACCCACTGAACCAAGCTCTA; *KIR2DL2* reverse: AGACTCTTGGTCCATTACCG; *KIR2DL3* forward: CTCATGGTCGTCAGCATGGT; *KIR2DL3* reverse: CTGTGCAGAAGGAAGTGCTG; β-actin forward: AAGATCATTGCTCCTCCTG; β-actin reverse: CGTCATACTCCTGCTTGCTG. The PCR amplification procedure was as follows: 10 s at 95°C followed by 40 cycles of 5 s at 95°C and 30 s at 64°C. Each standard and sample value was determined in three independent experiments. In each experiment, *KIR2DL2* and *KIR2DL3* expression under each experimental condition was calculated using threshold cycle (Ct) values standardized to β-actin (housekeeping gene), applying the 2^−(ΔCt)^ method (35).

### Western Blot Analysis

Protein samples from γδ T cells treated with DAC and mithramycin (Sigma-Aldrich) were homogenized using RIPA lysis buffer. Equal amounts of protein per sample were separated using SDS-PAGE gel and transferred to polyvinylidene fluoride membranes. The membranes were incubated for 1 h with blocking buffer containing 5% skimmed milk, and then incubated with the primary Abs for KIR2DL2, KIR2DL3, and rabbit anti-goat or goat anti-mouse IgG Ab (R&D Systems). An ECL detection system was used to visualize the proteins.

### Statistical Analysis

Data were analyzed by paired *t*-test or one-way ANOVA using GraphPad Prism (GraphPad Software Inc., La Jolla, CA, USA). The comparative *C*T method was applied in the quantitative real-time RT-PCR assay according to the delta-delta *C*T method (36). Results are presented as means ± SD and considered significant at a *p* < 0.05.

## Results

### DAC Induced Negligible Apoptosis of γδ T Cells, but Significantly Inhibited γδ T Cell Proliferation and Cytotoxicity

**Figure 1:**
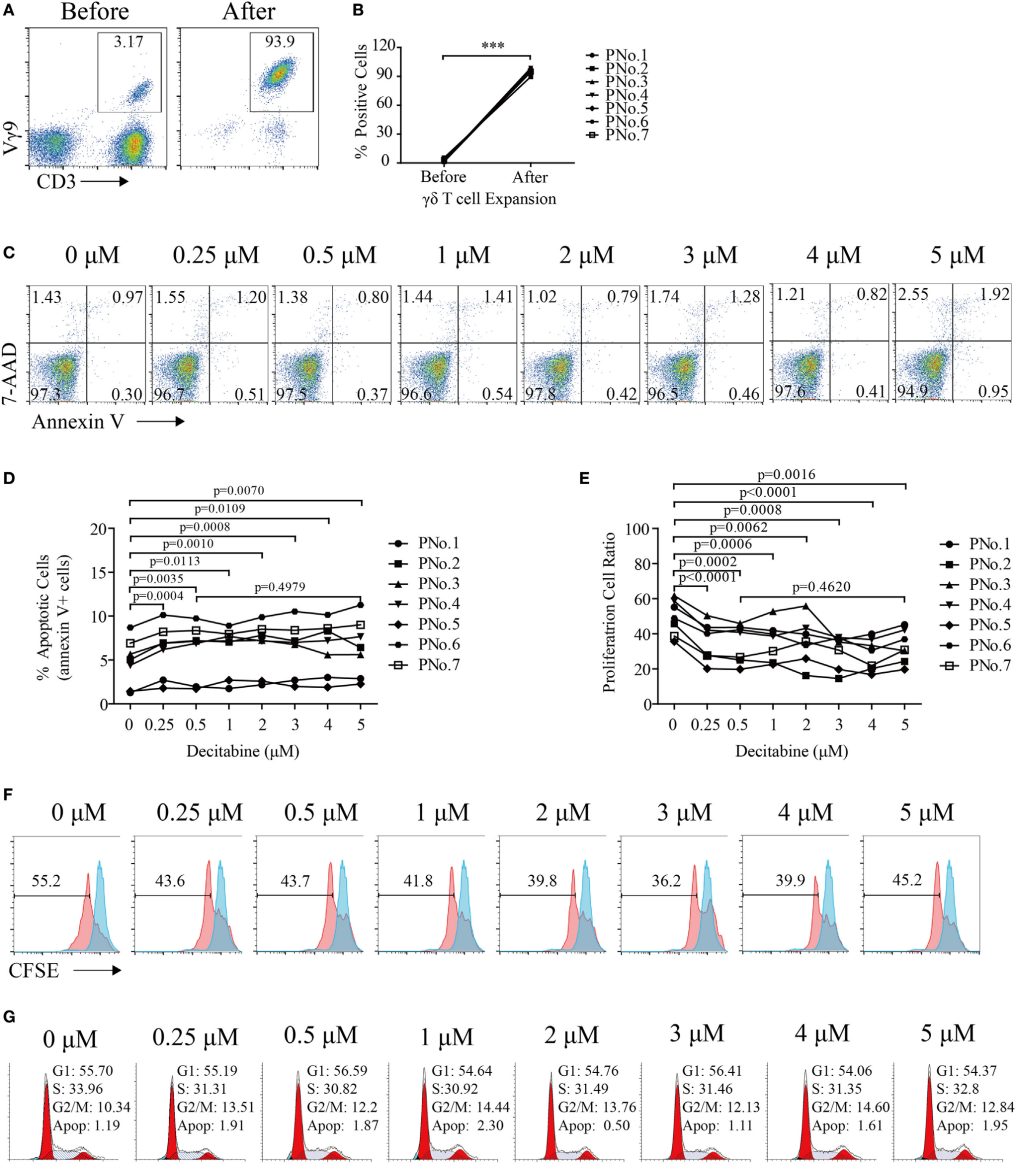
Decitabine inhibited the proliferation of gamma delta (γδ) T cells. **(A)** Representative flow cytometry analysis of CD3^+^Vγ9^+^ γδ T cells before and after expansion. **(B)** Percentage of γδ T cells before and after expansion (***p < 0.001; *n* = 7). **(C)** Viability of expanded γδ T cells after exposure to various DAC concentrations for 48 h. One representative experiment is shown. **(D)** Graph showing the apoptosis of expanded γδ T cells from seven patients after DAC treatment. The apoptosis percentages are shown as the percentage of cells that were annexin-V-positive. **(E)** Graph showing the proliferation of γδ T cells treated with increasing doses of DAC for 5 days. **(F)** Proliferation of γδ T cells treated with increasing doses of DAC. The red peaks represent the results of carboxyfluorescein succinimidyl ester (CFSE)-labeled γδ T cells after treatment with increasing concentrations of DAC for 5 days. The blue peak represents CFSE-labeled γδ T cells before DAC treatment. **(G)** Cell cycle of γδ T cells treated with increasing doses of DAC for 48 h.

γδ T cells were expanded from the PBMCs of seven MDS and AML patients. The percentage of γδ T cells in PBMCs before expansion was 2.65% (1.46–5.45%), whereas the percentage of γδ T cells after expansion was 95.2% (90–98.7%) (Figures 1A,B). After expansion, the mean fluorescence intensity (MFI) of Vγ9 increased from 10.5 (5.61–20.7) to 438 (389–569), which indicated that zoledronate plus IL-2 could increase the expression of Vγ9.

To assess the direct impact of DAC on γδ T cell viability, expanded γδ T cells were treated with various concentrations (from 0 to 5 µM) of DAC for 48 h. DAC can induce γδ T cell apoptosis (*p* < 0.05), although this effect is very weak. In addition, no difference in apoptosis between γδ T cells treated with 0.5 and 5 µM DAC was observed (*p* > 0.05) (Figures 1C,D).

To investigate the cytostatic effects of DAC on γδ T cells, CFSE dilution was detected after 5 days of DAC treatment in CFSE-labeled γδ T cells. We observed that DAC significantly inhibited γδ T cell proliferation. However, this effect did not change with increasing drug concentrations (Figures 1E,F). DAC had no effect on the cell cycle of γδ T cells (Figure 1G).

**Figure 2:**
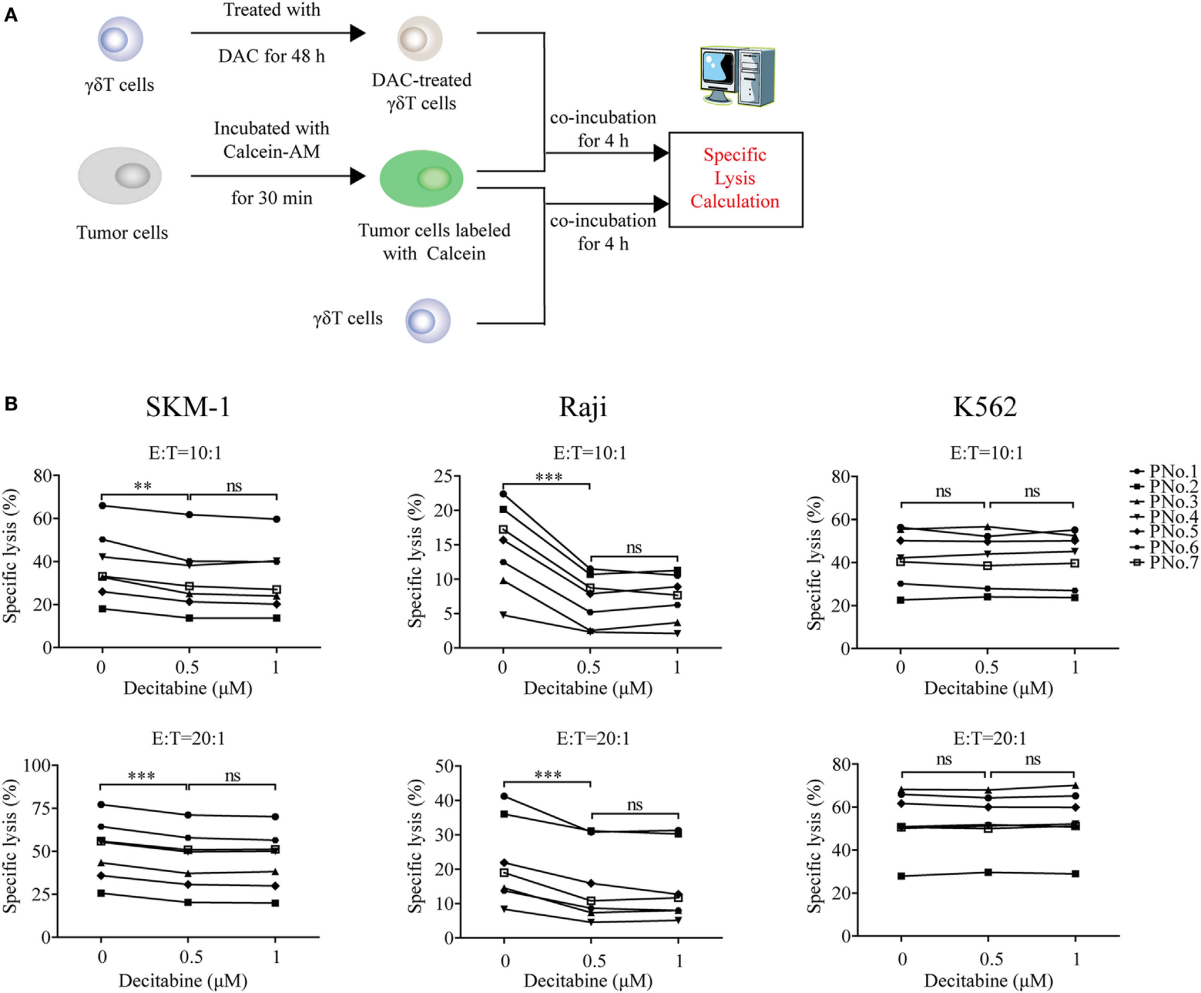

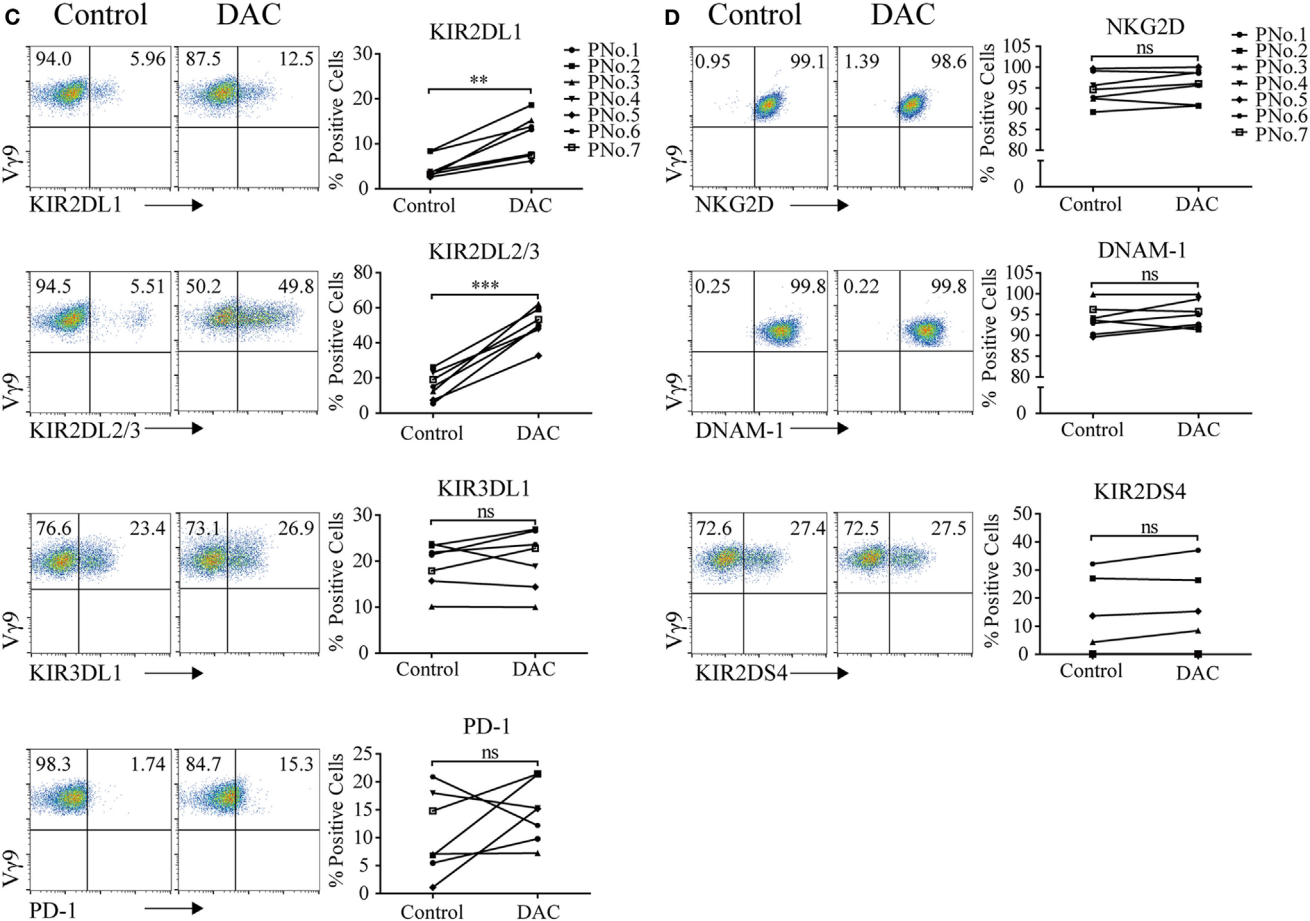
Decitabine treatment reduced gamma delta (γδ) T cell cytotoxicity to tumor cells and upregulated inhibitory receptor expression on γδ T cells. **(A)** Cytotoxicity assay protocol. **(B)** Cytotoxicity of expanded γδ T cells treated with increasing doses of DAC against Raji, K562, and SKM-1 cells in two effector to target cell (E:T) ratios (**p < 0.01; ***p < 0.001; ns, not significant; *n* = 7). **(C,D)** The effect of DAC on inhibitory and activating receptors expressed on γδ T cells. γδ T cells were incubated with 0.5 µM DAC for 48 h. Annexin-V-negative cells were gated and analyzed for surface marker expression (**p < 0.01; ***p < 0.001; ns, not significant; *n* = 7).

To evaluate whether DAC treatment weakened γδ T cell-mediated cytolysis of tumor cells, three kinds of tumor cells (MDS SKM-1 cells, NK-resistant Raji cells, and NK-sensitive K562 cells) were selected as target cells. The cytotoxicity assay was performed as shown in Figure 2A. The results indicated that γδ T cells treated with 0.5 µM DAC were significantly less cytotoxic to SKM-1 and Raji cells than untreated γδ T cells (0 µM) (*p* < 0.05). However, these effects did not change with increasing drug concentrations (*p* > 0.05). DAC treatment did not affect γδ T cell lysis of K562 cells (*p* > 0.05) (Figure 2B).

### DAC Inhibited γδ T Cell Cytotoxicity to Tumor Cells Through Upregulation of KIR2DL2/3 Expression on γδ T Cells

To investigate whether DAC affected the expression of inhibitory and activating receptors further, γδ T cells were treated with 0.5 µM DAC for 48 h. We found that DAC increased the expression of KIR2DL1 (CD158a) and KIR2DL2/3 (CD158b) on γδ T cells (*p* < 0.05). In some patients, DAC upregulated the expression of KIR3DL1 (CD158e1) and PD-1 on γδ T cells (Figure 2C). However, DAC did not affect the expression of activating receptor, including that of NKG2D, DNAM-1, and KIR2DS4 (*p* > 0.05) (Figure 2D).

**Figure 3:**
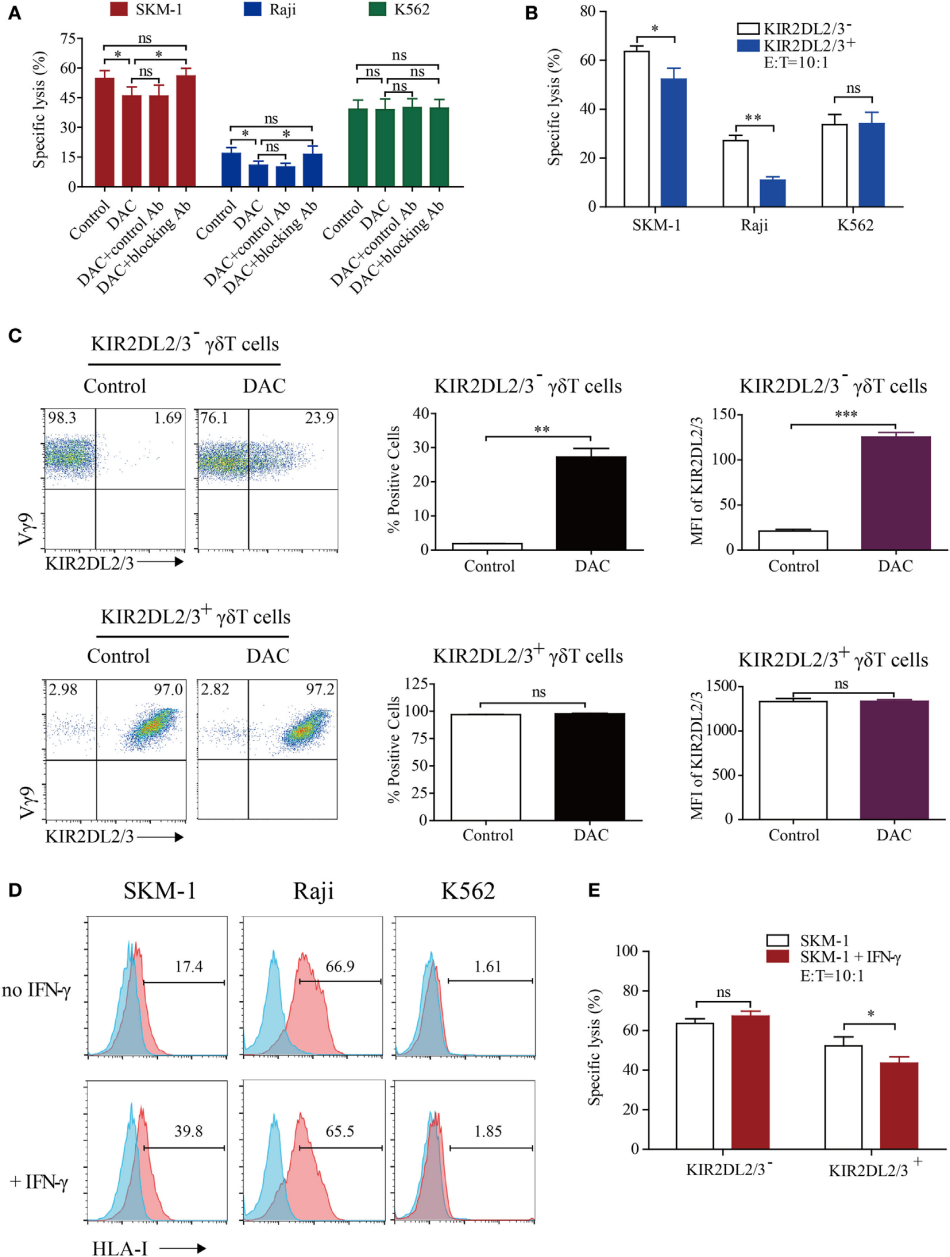
Decitabine induced polarization of KIR2DL2/3-negative gamma delta (γδ) T cells to KIR2DL2/3-positive cells, which are less cytotoxic to tumor cells expressing HLA-I molecules than KIR2DL2/3-negative γδ T cells. **(A)** The decrease in γδ T cell killing was inhibited by the KIR2DL2/3-blocking antibody (Ab). The 0.5 µM DAC-treated γδ T effector cells were first treated with or without Abs to KIR2DL2/3 for 30 min and then incubated with target cells at 37°C for 4 h to test the cytotoxicity (*p < 0.05; ns, not significant; *n* = 3). **(B)** The cytotoxicity of KIR2DL2/3-positive and -negative γδ T cells to tumor cells. KIR2DL2/3-positive and -negative γδ T cells were incubated with Raji, SKM-1, and K562 cells at 37°C for 4 h to test the cytotoxicity (*n* = 3; *p < 0.05; **p < 0.01; ns, not significant). **(C)** Effect of DAC on KIR2DL2/3 expression on KIR2DL2/3-positive and -negative γδ T cells. KIR2DL2/3-positive and -negative γδ T cells were sorted from cultured γδ T cells and treated with 0.5 µM DAC for 48 h. FACS dot plots showing the changes of KIR2DL2/3 on KIR2DL2/3-positive and -negative γδ T cells after treatment with DAC. Graph showing the percentage and mean fluorescence intensity (MFI) of KIR2DL2/3 on KIR2DL2/3-positive and -negative γδ T cells after treatment with DAC (**p < 0.01; ***p < 0.001; ns, not significant; *n* = 3). **(D)** HLA-I molecule expression on tumor cells and interferon (IFN)-γ-treated tumor cells. Tumor cells were first treated with 200 IU/mL IFN-γ for 48 h, and then harvested and stained with mouse anti-human HLA-I molecular Ab. **(E)** The cytotoxicity of KIR2DL2/3-positive and -negative γδ T cells to SKM-1 cells and IFN-γ-treated SKM-1 cells (*p < 0.05; ns, not significant; *n* = 3).

We next evaluated whether KIR2DL2/3 upregulation was relevant to the decreased cytotoxicity of DAC-treated γδ T cells. As can be seen in Figure 3A, DAC-treated γδ T cells showed a lower cytotoxicity to SKM-1 and Raji cells than γδ T cells (45.83 ± 8.01 vs. 54.65 ± 6. 97% and 10.86 ± 3.65 vs. 16.86 ± 5.05%; *n* = 3). However, there was no difference in cytotoxicity between γδ T cells and DAC-treated γδ T cells incubated with KIR2DL2/3-blocking Ab to SKM-1 (54.65 ± 6. 97 vs. 55.94 ± 6.71%; *n* = 3) and Raji (16.86 ± 5.05 vs. 16.34 ± 7.31%; *n* = 3) cells. These data indicated that KIR2DL2/3-blocking Ab completely restored the cytotoxicity of DAC-treated γδ T cells to SKM-1 and Raji cells. However, KIR2DL2/3-blocking Ab had no effect on the cytotoxicity of γδ T cells against K562 cells. These findings indicated that the decreased cytotoxicity of DAC-treated γδ T cells was dependent on the interaction between KIR2DL2/3 and their ligands (Figure 3A). In the blocking experiment, many γδ T cells are needed. Owing to insufficient number of γδ T cells, we performed the blocking experiment only at one effector to target cell ratio (10:1). Although only one effector to target cell ratio was measured in the blocking assay, the effector γδ T cells were obtained from three patients. We believe that the above data can support our statement.

To investigate whether the expression of KIR2DL2/3 was associated with the decreased cytotoxicity of DAC-treated γδ T cells further, we determined the cytotoxicity of KIR2DL2/3^+^ and KIR2DL2/3^−^ γδ T cells. γδ T cells were sorted by FACS. The purity of KIR2DL2/3-positive and -negative γδ T cells was above 95%. KIR2DL2/3^+^ γδ T cells were significantly less cytotoxic to SKM-1 and Raji cells than KIR2DL2/3^−^ γδ T cells (*p* < 0.05). In terms of cytotoxicity to K562 cells, there was no difference between KIR2DL2/3^+^ and KIR2DL2/3^−^ γδ T cells (*p* > 0.05) (Figure 3B). After incubation with 0.5 µM DAC for 48 h, some KIR2DL2/3^−^ γδ T cells expressed KIR2DL2/3. However, DAC did not affect KIR2DL2/3 expression on KIR2DL2/3^+^ γδ T cells (Figure 3C).

To investigate whether the decreased cytotoxicity of KIR2DL2/3^+^ γδ T cells to SKM-1 and Raji cells was associated with the HLA-C allotypes in tumor cells, we analyzed the HLA-C allotypes in SKM-1 and Raji cell lines through genotyping by Search Biotech Co., Ltd. (Beijing, China). The HLA-C alleles of SKM-1 cells are HLA-C*04:01 homozygotes and the HLA-C alleles of Raji cells are HLA-C*03:04 and C*04:01. Both HLA-C*03:04 and HLA-C*04:01 are ligands of KIR2DL2/3 (37, 38). We further determined the expression of HLA-I molecules on target tumor cells and found that HLA-I molecular expression level was low on SKM-1 cells, high on Raji cells, and very low on K562 cells (Figure 3D). Interferon (IFN)-γ has been reported to enhance HLA-I molecular expression on tumor cells. We found that IFN-γ only increased HLA-I molecular expression on SKM-1 cells, but not on Raji and K562 cells (Figure 3D). Meanwhile, compared to SKM-1 cells, KIR2DL2/3^+^ γδ T cells were less cytotoxic to IFN-γ-treated SKM-1 cells (*p* < 0.05). However, the cytotoxicity of KIR2DL2/3^−^ γδ T cells to IFN-γ-treated and untreated SKM-1 cells showed no difference (*p* > 0.05) (Figure 3E).

### Epigenetic Regulation of *KIR2DL2/3* Expression by DAC

**Figure 4:**
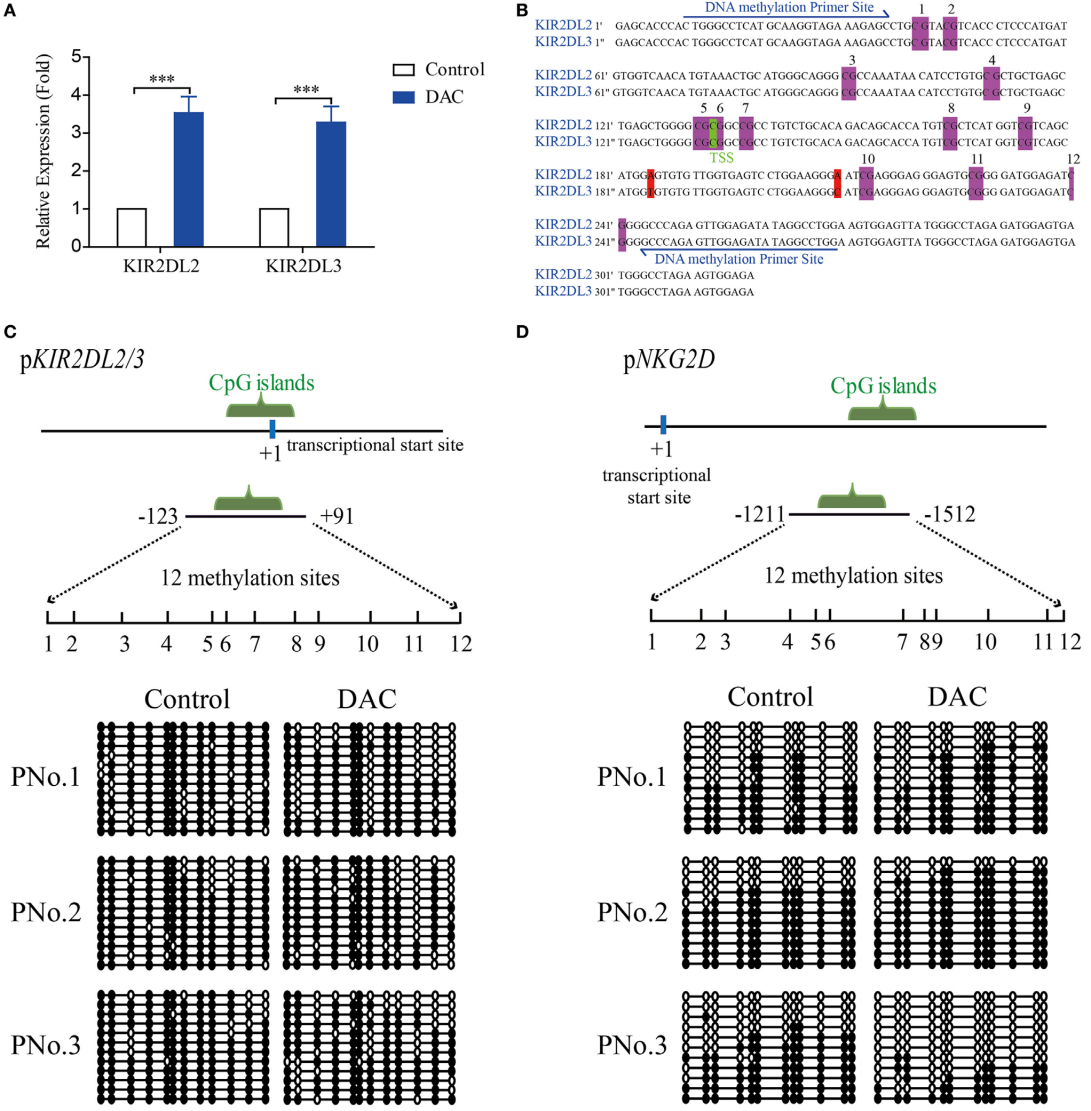

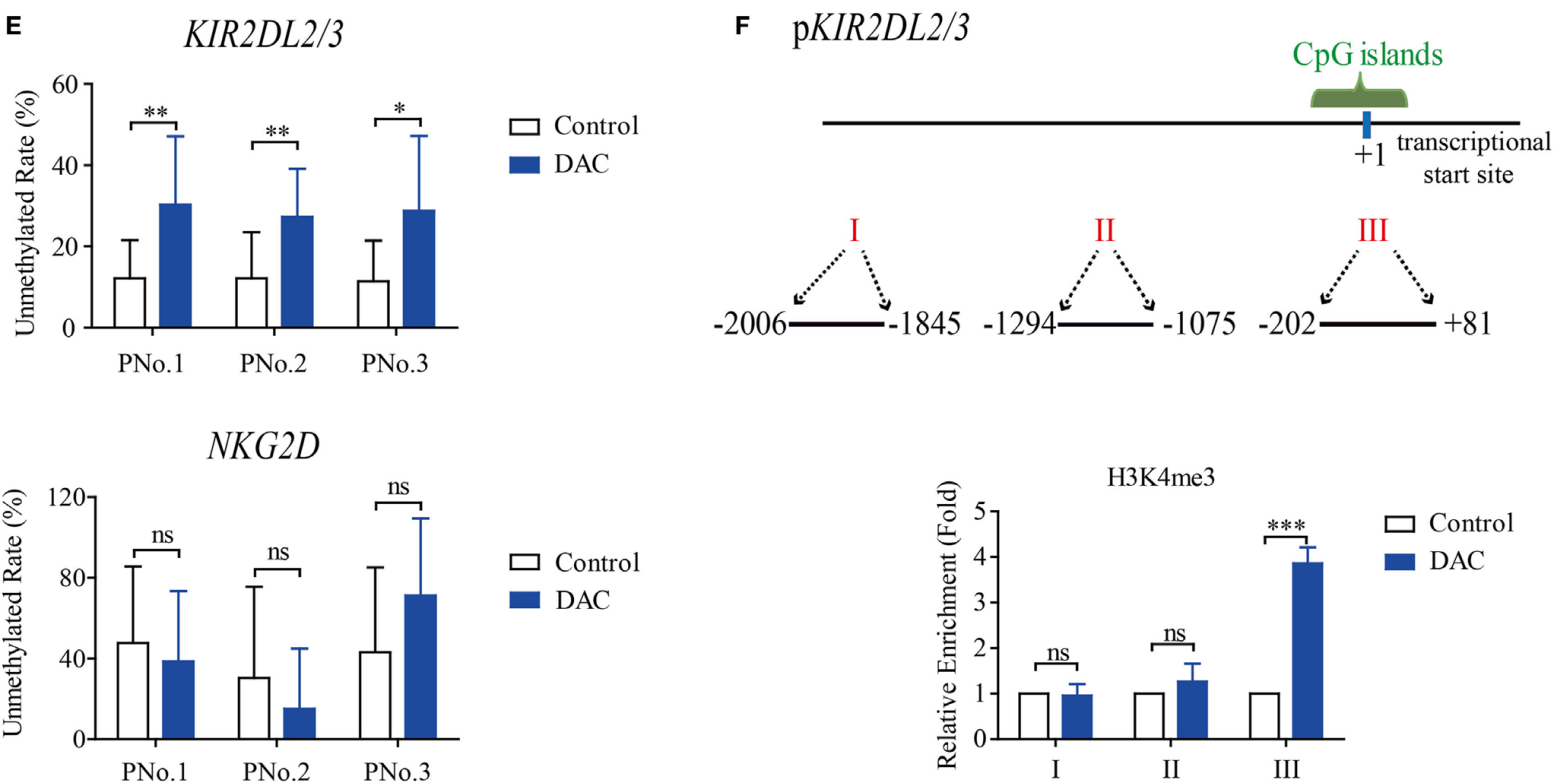
Decitabine increased demethylation of *KIR2DL2/3* promoter, which was beneficial to the transcription of *KIR2DL2/3* in gamma delta (γδ) T cells. **(A)** qRT-PCR analysis of *KIR2DL2* and *KIR2DL3* expression on γδ T cells exposed to 0.5 µM DAC for 48 h. Data are expressed as the expression of KIR2DL2 and KIR2DL3 mRNA on DAC-treated γδ T cells relative to that on γδ T cells (***p < 0.001; *n* = 3). **(B)** The genes around the transcriptional star sites (TSS) (marked green) of KIR2DL2 and KIR2DL3 are nearly the same, except for two bases (marked red). There are 12 CpG sites (marked purple) around the TSS of KIR2DL2 and KIR2DL3 genes. **(C,D)** DNA methylation of the promoters of both *KIR2DL2/3* and *NKG2D* in γδ T cells. The upper images show the schematic diagram of CpG islands in *KIR2DL2/3* and *NKG2D* promoters. Vertical lines: methylation sites. The lower images show DNA methylation of *KIR2DL2/3* and *NKG2D* promoters in γδ T cells treated with and without (control) DAC for 48 h from three patients. Open circles: unmethylated CpGs; solid circles: methylated CpGs. **(E)** Statistical analysis of the demethylation states of *KIR2DL2/3* and *NKG2D* promoters in γδ T cells from three patients (*p < 0.05; **p < 0.01; ns, not significant). **(F)** Three regions (I, II, and III) including TSS (+1) of *KIR2DL2/3* were analyzed by chromatin immunoprecipitation qPCR for H3K4me3 occupancy in the *KIR2DL2/3* promoter of γδ T cells treated with and without (control) DAC for 48 h. Enrichment of *KIR2DL2/3* promoter-specific DNA sequences was measured using quantitative PCR. Data are shown from three independent experiments and each PCR was performed in triplicate (***p < 0.001; ns, not significant).

To examine whether DAC treatment influenced the expression of KIR2DL2/3 at the transcriptional level, we examined the gene expression patterns of KIR2DL2/3 in γδ T cells. Real-time RT-PCR results indicated that DAC increased the expression of *KIR2DL2* and *KIR2DL3* (*p* < 0.05) (Figure 4A). The upregulation of KIR2DL2/3 expression was primarily due to the enhanced transcription of *KIR2DL2/3* gene. Therefore, we determined whether epigenetic mechanisms, such as DNA methylation, were involved in regulating *KIR2DL2* and *KIR2DL3* genes. We found that the genes around the transcriptional star sites (TSS) of *KIR2DL2* and *KIR2DL3* were highly consistent (Figure 4B), and there was a CpG island and 12 CpG sites around the TSS of *KIR2DL2/3* gene (between −123 and +91 positions), as predicted by the website of Li Lab (http://www.urogene.org/methprimer/) (Figure 4B). We analyzed the status of DNA methylation in the CpG islands of *KIR2DL2/3* in γδ T cells treated with and without DAC from three cancer patients by using bisulfite sequencing. As shown in Figures 4C,E, there was a significant enhancement of DNA demethylation in the *KIR2DL2/3* promoter of DAC-treated γδ T cells. However, DAC did not demethylate the *NKG2D* promoter (Figures 4D,E).

Histone H3K4 tri-methylation (H3K4me3) is positively correlated with transcriptional activity (39). We observed a significantly increased level of H3K4me3 around the CpG islands in DAC-treated γδ T cells compared with DAC-untreated γδ T cells (*p* < 0.05) (Figure 4F).

Altogether, the results indicated that demethylation in the *KIR2DL2/3* gene promoter of DAC-treated γδ T cells correlated with the enhanced expression of KIR2DL2/3.

### DAC Increased KIR2DL2/3 Expression Through Sp-1-Binding Activity

**Figure 5:**
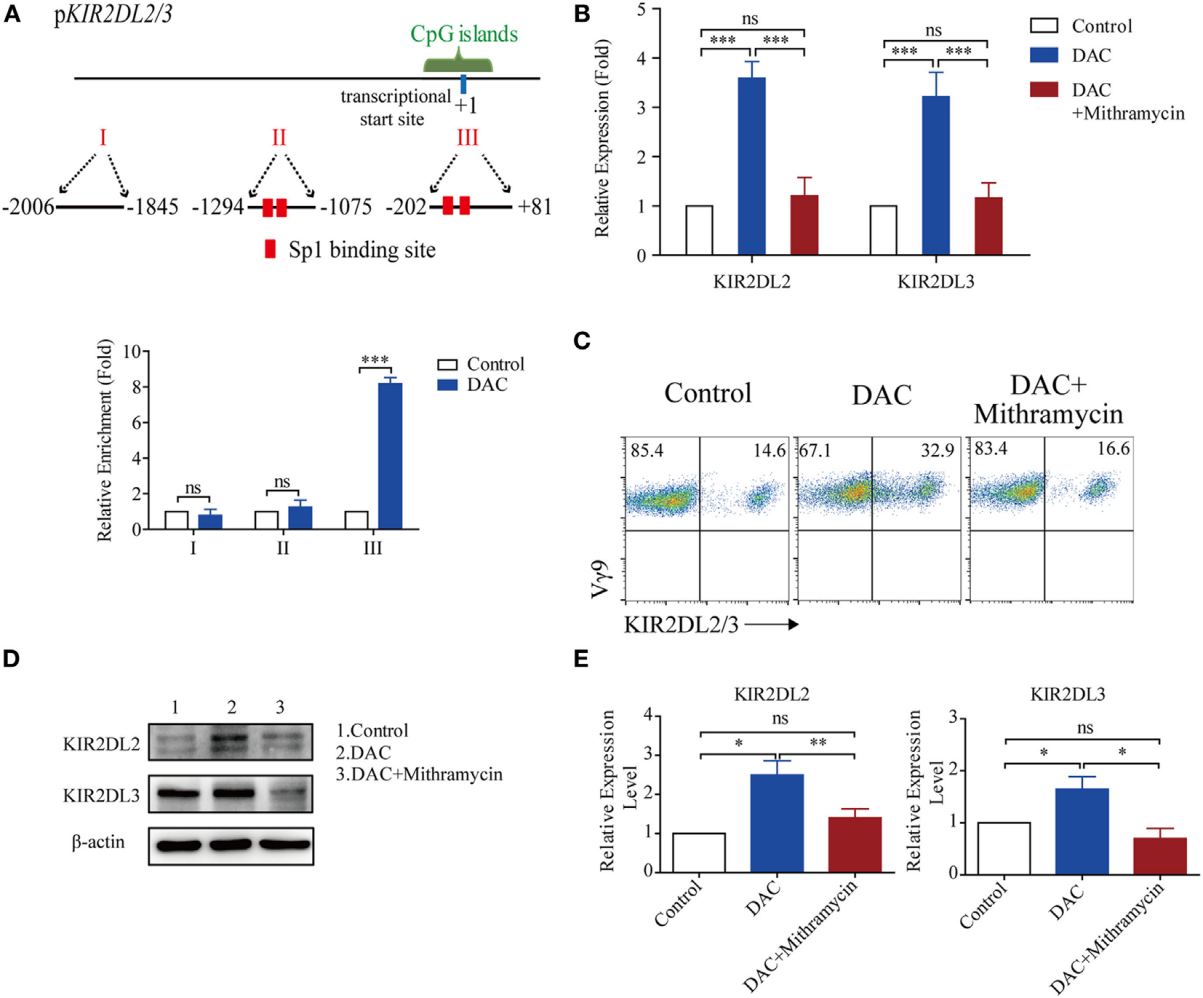
Decitabine increased KIR2DL2/3 expression by ligation of Sp-1 and its binding sites. **(A)** Three regions (I, II, and III) including transcriptional star site (+1) of *KIR2DL2/3* were analyzed by chromatin immunoprecipitation qPCR for Sp-1 occupancy in *KIR2DL2/3* promoter of gamma delta (γδ) T cells treated with and without (control) DAC for 48 h. Data are shown from three independent experiments and each PCR was performed in triplicate (***p < 0.001; ns, not significant). **(B)** The effect of the Sp-1-inhibitor mithramycin on *KIR2DL2* and *KIR2DL3* expression in DAC-treated γδ T cells. qRT-PCR analysis of *KIR2DL2* and *KIR2DL3* expression in γδ T cells, γδ T cells treated with 0.5 µM DAC and γδ T cells treated with 0.5 µM DAC and 100 µM mithramycin for 48 h. Data are expressed as the relative expression of KIR2DL2 and KIR2DL3 mRNA in different groups (***p < 0.001; ns, not significant; *n* = 3). **(C)** Flow cytometry analysis of the effect of the Sp-1-inhibitor mithramycin on DAC-treated γδ T cells. γδ T cells were treated with 0.5 µM DAC and 0.5 µM DAC plusing 100 µM mithramycin for 48 h, respectively. Representative FACS results illustrate KIR2DL2 and KIR2DL3 expression on γδ T cells, DAC-treated γδ T cells, and γδ T cells treated with both DAC and mithramycin. **(D)** Western blot analysis of the effect of mithramycin on DAC-treated γδ T cells. γδ T cells were treated with 0.5 µM DAC and 0.5 µM DAC plusing 100 µM mithramycin for 48 h, respectively. A representative western blot illustrates KIR2DL2 and KIR2DL3 expression on γδ T cells, DAC-treated γδ T cells, and γδ T cells treated with both DAC and mithramycin. β-Actin was used as a loading control of the cell lysates. **(E)** Bands of KIR2DL2 and KIR2DL3 were quantified by densitometric analysis and plotted after normalization against β-actin. The histogram shows means ± SD for three independent sets of experiments (*p < 0.05; **p < 0.01; ns, not significant).

There were two Sp-1 transcription factor potential binding sites in the CpG islands of the *KIR2DL2/3* promoter (Figure 5A). Therefore, we proceeded to investigate whether DAC affected the binding of Sp-1 to its potential binding sites, to upregulate the expression of *KIR2DL2/3* in γδ T cells. The chromatin from γδ T cells treated with and without DAC was immunoprecipitated with anti-Sp-1 Ab. The immunoprecipitated samples were subjected to qPCR using primers to specifically amplify the CpG islands. Another two pairs of primers were also used to amplify the regions in the *KIR2DL2/3* promoter, except the CpG islands. The qPCR results indicated that DAC substantially increased the binding of Sp-1 to its potential binding sites in the CpG islands of the *KIR2DL2/3* promoter (*p* < 0.05) (Figure 5A).

To ascertain whether DAC increased KIR2DL2/3 expression through the ligation of Sp-1 and its binding sites in the CpG islands of the *KIR2DL2/3* promoter, the Sp-1 inhibitor mithramycin was added to γδ T cells treated with DAC. Mithramycin prevented *KIR2DL2* and *KIR2DL3* upregulation mediated by DAC in γδ T cells (Figure 5B). Flow cytometry and western blot assay indicated that mithramycin substantially inhibited the upregulated KIR2DL2/3 expression induced by DAC (Figures 5C–E). These findings indicated that DAC enhanced KIR2DL2/3 expression in γδ T cells by increasing the binding of Sp-1 to its binding sites in the CpG islands of the KIR2DL2/3 promoter.

## Discussion

DAC has been approved for the treatment of hematological malignancies, and its clinical effects on solid tumors have gained attention (17). It also affects immune cells. In a previous study, DAC induced FOXP3 hypomethylation and increased the number of regulatory T cells (30). Another study indicated that DAC inhibited naïve T cell proliferation by increasing the gene expression of the DNA dioxygenase TET2, which facilitated the expression of several cell cycle inhibitors (31). The effects of DAC on NK cells are controversial. Benjamin et al. reported that DAC augmented IFN-γ secretion by NK cells as well as cytotoxicity to tumor cells (40). However, another report claimed that the same dose of DAC treatment resulted in the inhibition of proliferation and decreased cytotoxicity of NK cells (20). It has been reported that DAC treatment resulted in a rapid and stable induction of transcription and cell surface expression of KIR2DL2/3 in NK cell lines, NK cell clones, and freshly isolated NK cells, but not in the T cell line, Jurkat, or the B cell line RPMI 8866 (41). In this study, we demonstrated that the proliferation of γδ T cells was inhibited by DAC. However, DAC had no effect on the cell cycle of γδ T cells. DAC significantly upregulated the expression of KIR2DL2/3, thereby reducing γδ T cell cytotoxicity to tumor cells expressing the ligand of KIR2DL2/3.

KIR2DL2/3 is a member of the inhibitory killer Ig-like receptors (KIRs), which recognize a defined group of polymorphic HLA-I molecules. KIR2DL2/3 inhibits NK cell-mediated lysis of target cells bearing the appropriate HLA-I allotypes through an immune tyrosine-based inhibitory motif by recruiting protein tyrosine phosphatases (SHP-1 and SHP-2) (42–45). Furthermore, the combination of KIR2DL2/3 and their ligands seems to favor NK cell inhibition and malignant melanoma dissemination (46). γδ T cells display MHC-independent cytotoxicity against various tumors (2). However, we observed that KIR2DL2/3^+^ γδ T cells were less cytotoxic to tumor cells expressing HLA-I molecules than KIR2DL2/3^−^ γδ T cells. This result suggests that the lytic activity of γδ T cells against tumor cells was influenced by signaling *via* KIR2DL2/3.

KIR2DL2/3 protein contains KIR2DL2 and KIR2DL3. We found that the promoter regions of *KIR2DL2* and *KIR2DL3* are highly similar, with more than 99% DNA sequence identity. CpG islands were present in areas surrounding the transcription initiation region of both *KIR2DL2* and *KIR2DL3*, and the DNA sequence was highly consistent. It has been reported that CpG islands surrounding the transcriptional start site of each KIR gene are consistently demethylated in expressed KIR and methylated in unexpressed KIR (41). Our data indicated that DAC treatment demethylated *KIR2DL2/3* genes in γδ T cells and enriched H3K4me3 in the CpG islands of *KIR2DL2/3* gene. The enrichment of H3K4me3 near the transcription start site defined an active state of gene expression (47, 48). Therefore, our data suggested that DAC increased KIR2DL2/3 expression in γδ T cells through an epigenetic mechanism.

Previous studies have shown that transcription factors such as Sp-1 play an important role in KIR gene expression (49–51). They can act as both negative and positive regulators of gene expression depending on their relative concentration and binding partners (52, 53). Through the prediction of transcription factor-binding sites using the AliBaba 2.1 website, we found that there were two Sp-1-binding sites in the CpG islands of *KIR2DL2/3* gene. We noticed a significant enrichment of Sp-1 in the CpG islands of *KIR2DL2/3* gene in DAC-treated γδ T cells compared with DAC-untreated γδ T cells. Mithramycin A, which inhibits the interaction of Sp-1 with its binding site (54–56), significantly attenuated KIR2DL2/3 expression in DAC-treated γδ T cells. These results indicated that DAC enhanced KIR2DL2/3 expression through the enrichment of Sp-1 binding to its binding sites.

**Figure 6:**
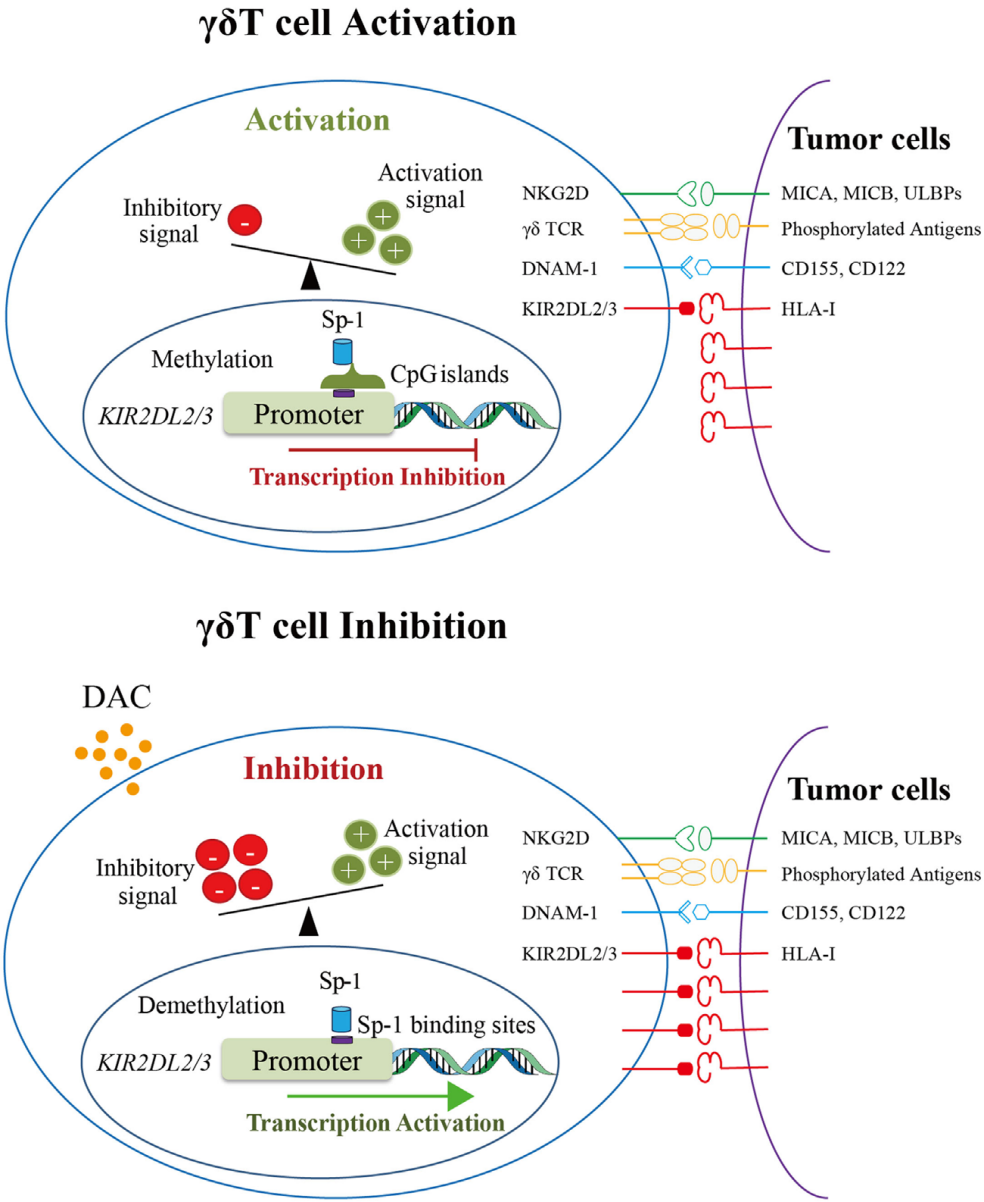
The proposed mechanism underlying the inhibition of gamma delta (γδ) T cell cytotoxicity by decitabine *via* promoting KIR2DL2/3 expression. The upper image shows the schematic diagram of γδ T cell activation. KIR2DL2/3 expression on γδ T cells was very low because the promoter of KIR2DL2/3 gene was methylated. When encountering tumor cells, γδ T cells were activated by the recognition of activation receptors and their ligands on tumors cells. The lower image shows the schematic diagram of γδ T cell inhibition by DAC. DAC demethylates the promoter of *KIR2DL2/3*, which increases the binding of Sp-1 to its potential binding sites in the *KIR2DL2/3* promoter, and then activates KIR2DL2/3 gene and protein expression. The upregulation of KIR2DL2/3 protein expression reduces the cytotoxicity of γδ T cells against tumor cells expressing HLA-I molecules.

We suggest that in activated γδ T cells, the activation signal was much stronger than the inhibitory signal when γδ T cells encountered tumor cells expressing HLA-I molecules because of the methylation of *KIR2DL2/3* as well as low expression level of KIR2DL2/3. However, DAC can demethylate *KIR2DL2/3* genes of γδ T cells and promote the binding of Sp-1 to *KIR2DL2/3* gene promoter, which consequently promotes *KIR2DL2/3* gene transcription and KIR2DL2/3 protein expression. When encountering tumor cells expressing HLA-I molecules, the cytotoxicity of DAC-treated γδ T cells, which have a higher level of KIR2DL2/3 expression, is decreased (Figure 6).

Our study reveals that DAC-treated γδ T cells from all patients have a high level of KIR2DL2/3 expression and are less cytotoxic than DAC-untreated γδ T cells. Furthermore, we elucidated the underlying mechanism. These observations broadened our knowledge of the effect of DAC on γδ T cells. Because of the inhibitory effect of DAC on γδ T cell cytotoxicity, it is necessary to consider this side effect of DAC before using it in the clinical setting. Therefore, we recommend the monitoring of DAC concentration in patients’ blood after DAC treatment. After DAC clearance by the human body, adoptive γδ T cell infusion might be a good method for replenishing γδ T cells affected by DAC in the human body.

## Ethics Statement

Seven newly diagnosed MDS and AML patients who did not receive radiation therapy and chemotherapy before blood collection were enrolled in this study, all of whom provided with written informed consent for the use of biospecimens for research purposes in accordance with the Declaration of Helsinki. The study was approved by the Ethics Committee of the First Hospital of Jilin University and carried out in accordance with the approved guideline “Use of experimental animals and human subjects.”

## Author Contributions

JWC, JTC, YL, and WL conceived and designed the research. CN, ML, SZ, YC, and LZ performed the experiments and analyzed the data. DX contributed to sample collection. CN and SZ wrote the paper. All authors reviewed the manuscript.

## Conflict of Interest Statement

The authors declare no potential conflicts of interest.
